# Training with Hybrid Assistive Limb for walking function after total knee arthroplasty

**DOI:** 10.1186/s13018-018-0875-1

**Published:** 2018-07-03

**Authors:** Kenichi Yoshikawa, Hirotaka Mutsuzaki, Ayumu Sano, Kazunori Koseki, Takashi Fukaya, Masafumi Mizukami, Masashi Yamazaki

**Affiliations:** 1 Department of Physical Therapy, Ibaraki Prefectural University of Health Sciences Hospital, 4773 Ami, Ami-machi, Inashiki-gun, Ibaraki, 300-0331 Japan; 2 Department of Orthopaedic Surgery, Ibaraki Prefectural University of Health Sciences, 4669-2 Ami, Ami-machi, Inashiki-gun, Ibaraki, 300-0394 Japan; 3 Department of Physical Therapy, Faculty of Health Sciences, Tsukuba International University, 6-8-33 Manabe, Tsuchiura, Ibaraki, 300-0051 Japan; 4 Department of Physical Therapy, Ibaraki Prefectural University of Health Sciences, 4669-2 Ami, Ami-machi, Inashiki-gun, Ibaraki, 300-0394 Japan; 5 Department of Orthopaedic Surgery, Faculty of Medicine, University of Tsukuba, 1-1-1 Tennodai, Tsukuba, Ibaraki, 305-8575 Japan

**Keywords:** Total knee arthroplasty, Osteoarthritis, Rheumatoid arthritis, Robot assisted training, Hybrid assistive limb

## Abstract

**Background:**

The Hybrid Assistive Limb (HAL, CYBERDYNE) is a wearable robot that provides assistance to patients while walking, standing, and performing leg movements based on the intended movement of the wearer. We aimed to assess the effect of HAL training on the walking ability, range of motion (ROM), and muscle strength of patients after total knee arthroplasty (TKA) for osteoarthritis and rheumatoid arthritis, and to compare the functional status after HAL training to the conventional training methods after surgery.

**Methods:**

Nine patients (10 knees) underwent HAL training (mean age 74.1 ± 5.7 years; height 150.4 ± 6.5 cm; weight 61.2 ± 8.9 kg), whereas 10 patients (11 knees) underwent conventional rehabilitation (mean age 78.4 ± 8.0 years; height 150.5 ± 10.0 cm; weight 59.1 ± 9.8 kg). Patients underwent HAL training during 10 to 12 (average 14.4 min a session) sessions over a 4-week period, 1 week after TKA. There was no significant difference in the total physical therapy time including HAL training between the HAL and control groups. Gait speed, step length, ROM, and muscle strength were evaluated.

**Results:**

The nine patients completed the HAL training sessions without adverse events. The walking speed and step length in the self-selected walking speed condition, and the walking speed in the maximum walking speed condition were greater in the HAL group than in the control group at 4 and 8 weeks (*P* < 0.05). The step length in the maximum walking speed condition was greater in the HAL group than in the control group at 2, 4, and 8 weeks (*P* < 0.05). The extension lag and knee pain were lower in the HAL group than in the control group at 2 weeks (*P* < 0.05). The muscle strength of knee extension in the HAL group was greater than that in the control group at 8 weeks (*P* < 0.05).

**Conclusion:**

HAL training after TKA can improve the walking ability, ROM, and muscle strength compared to conventional physical therapy for up to 8 weeks after TKA. Since the recovery of walking ability was earlier in the HAL group than in the control group and adverse events were not observed in this pilot study, HAL training after TKA can be considered a safe and effective rehabilitation intervention.

**Trial registration:**

UMIN, UMIN000017623. Registered 19 May 2015

## Background

Total knee arthroplasty (TKA) is one of the most common surgeries for severe osteoarthritis (OA) and rheumatoid arthritis (RA) [1, 2]. Although it is possible to eventually obtain higher physical function and quality of life (QOL) through rehabilitation [3], physical function decreases immediately after TKA [4–6]. Moreover, studies suggest that walking speed and walking ability require about 1 year of recovery after TKA [6, 7]. In addition, knee extension lag occurs early after surgery, and restriction on range of active extension is observed [8–10]. Therefore, gaining improved walking function efficiently is particularly important for patients after TKA.

Robot-assisted training (RAT) has been developed since the early 2000s. There are several reports demonstrating improvements in walking ability through the application of RAT in patients with central nervous system diseases, such as stroke [11], spinal cord injury [12], and cerebral palsy [13]. However, there are few reports that confirm the effect of RAT in postoperative rehabilitation after TKA [14].

The Hybrid Assistive Limb (HAL, Cyberdyne Corporation, Tsukuba, Japan) is an assisted training device. HAL is a wearable robot that interactively provides motion according to the wearer’s voluntary drive [15]. Details of the HAL system have been reported in preliminary studies [16, 17]. Both single leg and two leg versions of the HAL are available, and the choice depends on the wearer’s requirements. The HAL detects the bioelectrical signals generated by the wearer’s muscle activity or the floor-reaction-force signals caused by the wearer’s weight shifts, or both. HAL enables lower limb exercise and gait training with voluntary drive, and has the advantages of both voluntary drive and ambulatory performance. Most other exoskeleton devices use autonomously generated predefined motion. In contrast, HAL provides motion in response to the wearer’s voluntary drive. The wearer operates HAL by adjusting his or her own muscle activity. Clinical trials of training using HAL have been already conducted for patients with stroke [18, 19], spinal cord injury [20], cerebral palsy [21], and neuromuscular diseases [22], and its clinical safety has been confirmed.

The study by Tanaka et al. [23] has been the only randomized controlled trial that compared the lower limb function between groups of patients who underwent training with HAL and conventional therapy after TKA. Although actual measured values were not described in the article, an immediate improvement within 1 week was observed with the use of HAL. This study was a short-term evaluation and was conducted for up to 3 weeks after surgery, and did not examine the improvement in range of motion (ROM). Moreover, the two-legged HAL was used in this study. Furthermore, the patients in this study had OA only, and RA was not included. The authors suggested that the single leg HAL may be better for functional recovery after TKA owing to its lighter weight and because it allows freedom of movements in the unaffected leg. Yoshioka et al. [24] showed an improvement of postoperative extension lag using a single joint type HAL (HAL-SJ), which is equipped with only one actuator to the knee joint motion for TKA patients, and Fukaya et al. [25] reported an improvement of postoperative walking ability in a case report of a single leg version of HAL. We hypothesized that the assistive benefit of HAL also reduces knee extension lag and improves walking ability and pre-operative function of TKA patients not only with OA but also with RA compared with conventional physical therapy.

Therefore, the purpose of this study was to evaluate the effect of training using the single leg version of HAL on walking ability, knee ROM, muscle strength, pain, and physical function, and to compare the functional status after HAL training with the conventional therapy alone and with the pre-operative functional status for up to 8 weeks after TKA for OA and RA.

## Methods

### Subjects

All subjects were admitted to our hospital between February 2015 and January 2018. Patients diagnosed with severe OA and RA via varus deformation of the knee by X-ray examination and who underwent primary TKA by a senior surgeon (H.M.) were included in the study. During this period, a total of 21 consecutive patients (23 knees) underwent primary TKA. The patients were categorized into two groups before undergoing surgery (HAL and control groups). The patients who agreed to receive HAL training and their physique allowed for HAL device were in the HAL group, because the size of a single leg HAL was in size medium only at that time. The other patients were in the control group. The patients in the HAL group underwent HAL training (average 14.4 ± 5.9 min a session) and conventional physical therapy (60 to 80 min a day) during the HAL intervention period. The HAL training started 1 to 5 weeks after TKA (HAL intervention period). The total number of HAL interventions ranged from 10 to 12 during the 4-week period. The patients in the control group underwent only conventional physical therapy (60 to 120 min a day) after TKA during the HAL intervention period of the HAL group. There was no significant difference in the total physical therapy time including HAL training between both groups during the HAL intervention period. 5 weeks after TKA, the patients in the both groups underwent same physical therapy. The physical therapy details in the both groups are summarized in Table 1. Eleven patients (12 knees) were included in the HAL group, whereas 10 patients (11 knees) were in the control group. Among the 11 patients allocated to the HAL group, 2 patients withdrew consent. One of the two patients could not continue the study because of worsening of depression (6 times HAL sessions were done). Another patient withdrew, because it was troublesome to continue the study (6 times HAL sessions were done). Therefore, 9 patients (10 knees) underwent HAL training. Before this study, 4 and 2 patients in the HAL and control groups, respectively, had already undergone TKA on the contralateral side. The pre-operative characteristics and baseline values did not differ significantly between the two groups (Table 2).

**Table 1:** Details of physical therapy and HAL training protocol

	HAL group	Control group	*P* value
HAL training	Single leg version HAL (size M)	None	
	1 week to 5 weeks after TKA (4 weeks)		
	Number of sessions: 11.6 ± 0.8		
	Average duration of session: 14.4 ± 5.9 min		
	●Knee ROM exercises (less than 20 min)		
	●Gait training (less than 20 min)		
Conventional PT	1 day after TKA to hospital discharge		
	5 or more days a week		
	60 to 80 min a day (during the HAL intervention period)	60 to 120 min a day (during the HAL intervention period)	
	●CPM: from 3 days after TKA		
	●FWB: from 1 week after TKA		
	●Gait training (flat ground, outdoor, irregular terrain)		
	●Stair climbing training		
	●Joint ROM training		
	●Muscle strengthening		
	●Balancing training (sitting and standing positions)		
	●ADL training (toilet, bathing, bedside tasks, etc.)		
	●Bicycle ergometer training		
	●Various physical exercises for returning to work		
	●Self-exercise education		
Total PT time including HAL training during HAL intervention period	26.5 ± 4.2 (h)	28.2 ± 5.2 (h)	0.434

Values are expressed as numbers or as mean ± SD

*Abbreviation*: *TKA*, total knee arthroplasty; *ROM*, range of motion; *PT*, physical therapy; *CPM*, continuous passive movement; *FWB*, full weight-bearing; *ADL*, activities of daily living

**Table 2:** Preoperative baseline characteristics of subjects

Characteristics		HAL group 9 patients (10 knees)	Control group 10 patients (11 knees)	*P* value
Age		74.1 ± 5.7	78.4 ± 8.0	0.180
Sex	Male/female	1/8	2 / 8	1.000
Weight (kg)		61.2 ± 8.9	59.1 ± 9.8	0.612
Height (m)		150.4 ± 6.5	150.5 ± 10.0	0.985
BMI (kg/m^2^)		27.1 ± 3.9	26.3 ± 5.3	0.719
Disease	OA/RA	8/2	10/1	0.587
TKA operated side	right/left	6/4	5/6	0.670
Contralateral side TKA		4	2	0.350

Values are expressed as numbers or as mean ± SD

Abbreviation: *BMI*, body mass index; *OA*, osteoarthritis; *RA*, rheumatoid arthritis; *TKA*, total knee arthroplasty

The ethics committee of Ibaraki Prefectural University of Health Sciences approved the study (no. e155). We explained the purpose of the study to the patients in verbal and written forms, and written consent was obtained from all patients. The protocol of this study was registered in the University Hospital Medical Information Network Clinical Trials Registry (UMIN000017623).

### Surgical procedure

The surgical procedure was similar to that described in our previous report [26]. Under general anesthesia, a midline skin incision was made, and a medial parapatellar approach was used. The patella was not replaced, and the posterior cruciate ligament was retained. The implants used were the NexGen® or Persona® (Zimmer, Warsaw, IN, USA) for the femoral component and the NexGen CR Stem Tibia or NexGen Trabecular Metal Monoblock Tibia (Zimmer, Warsaw, IN, USA) for the tibial component.

### HAL training

**Fig. 1:**
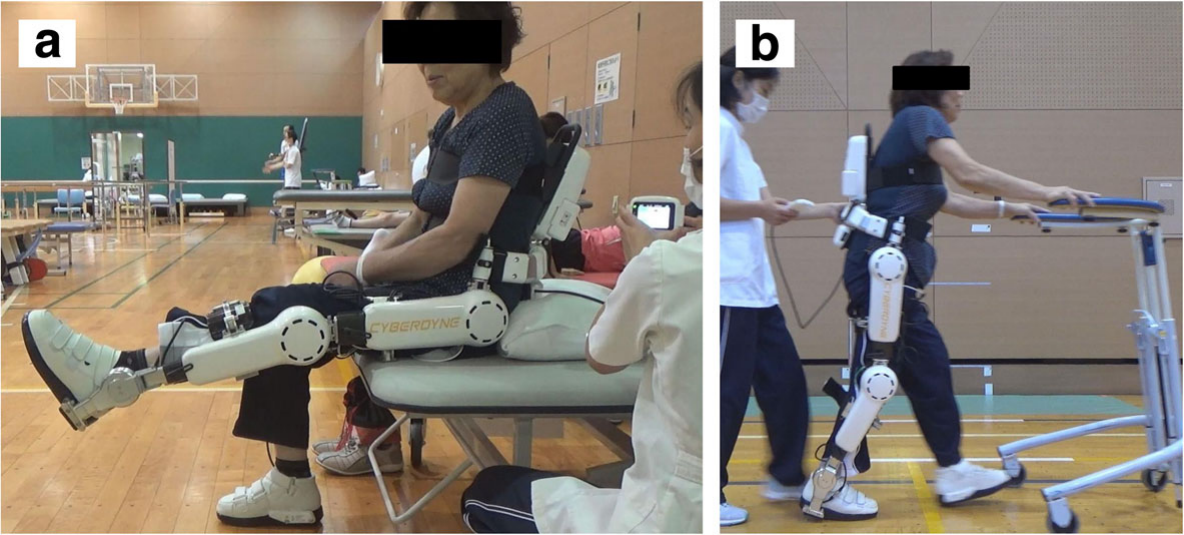
Hybrid Assistive Limb (HAL) training. Knee extension and flexion exercise while sitting, with HAL on the sagittal plane (**a**). Gait training, with HAL on the sagittal plane (**b**)

The single leg version of the HAL (size medium) (Table 1) was placed on the operative side, and the Cybernic Voluntary Control (CVC) mode was used. The gain in assistive torque at each joint in response to the bioelectrical signals was controlled by a therapist so that the patient could move the knee joint sufficiently and easily within the ROM without aggravating pain or presence of pain and extension lag, and the walk pattern was as normal and symmetrical as possible. First, repetitive knee flexion/extension exercises within the range where pain was not aggravated or was absent were performed for less than 20 min with the patient in the sitting position. Second, gait training with HAL was conducted on a level ground at a speed that the subject was comfortable with while still maintaining good gait posture, as judged by a physical therapist, for less than 20 min. To prevent falls, a wheeled walker was used during gait training with HAL (Fig. 1).

### Outcome measures

Indicators related to walking ability were as follows: self-selected walking speed (SWS) [27]; maximum walking speed (MWS) [27]; mean step length at SWS (SL-SWS) and MWS (SL-MWS); and cadence at SWS and MWS. SWS and MWS were measured according to the time taken to cover the intermediate 10 m of a total distance. Measurement was performed as many times as possible, up to a maximum of three times, and the fastest time was used. To calculate SL and cadence, the number of steps in the 10-m measurement section of the MWS or SWS test was counted. These were evaluated before the surgery and at weeks 2, 4, and 8.

The assessments related to knee function were as follows: ROM of knee flexion and extension motion on passive and active movements on the operated side; torque of knee extension and knee flexion as muscle strength of quadriceps and hamstrings; and Western Ontario and McMaster Universities Osteoarthritis Index (WOMAC) [28]. WOMAC validated for the Japanese patients who had TKA surgery was divided into subscales of pain (WOMAC-p) and physical function (WOMAC-f) [29]. ROM was measured by using a medical goniometer in 5 ° increments with the patients in the sitting position or the supine position. Torques of knee were measured with an isometric mode using Biodex System 4 (Biodex Medical Systems, NY, USA). The knee joint was fixed in 60 ° of knee flexion, and the maximum knee joint flexion and extension torque were measured. Both torques were measured in 3 sets every 5 s, and peak torque values were used. The value obtained by dividing the peak torque value by each body weight was used for analysis. ROM, both torques, and both WOMAC subscale scores were measured before the surgery and at weeks 2, 4, and 8. Four kinds of ROM were measured before the surgery and at weeks 1, 2, 3, 4, and 8.

### Statistical analysis

Differences in pre-operative subject characteristics were analyzed using a Student’s *t* test or the Wilcoxon’s rank sum test, as appropriate, for continuous variables, and Fisher’s exact test for categorical variables. For all outcome measures, two-way mixed analysis of variance (ANOVA) with repeated measures factor (from before surgery to week 8) and between-subjects factor (HAL group or control group) were performed for assessing 2 factor’s main effects and interaction between the 2 factors. If main effects of the repeated measures factor (time effect) or interactions satisfied significant level (*P* < 0.05) or significant large effect size (*η*_*p*_^2^ > 0.14), Tukey’s honestly significant difference was used for within-group comparisons of the outcomes at the various assessment time points [30]. If main effects of the between-subjects factor (intervention effect) or interactions satisfied a significant level (*P* < 0.05) or significant large effect size (*η*_*p*_^2^ > 0.14), Student’s *t* test was used for comparison between groups at the various assessment time points [30]. All analyses were performed with IBM SPSS Statistics version 24.0 (International Business Machines Corporation, Chicago, USA). Level of significance was set at *P* < 0.05.

## Results

**Fig. 2:**
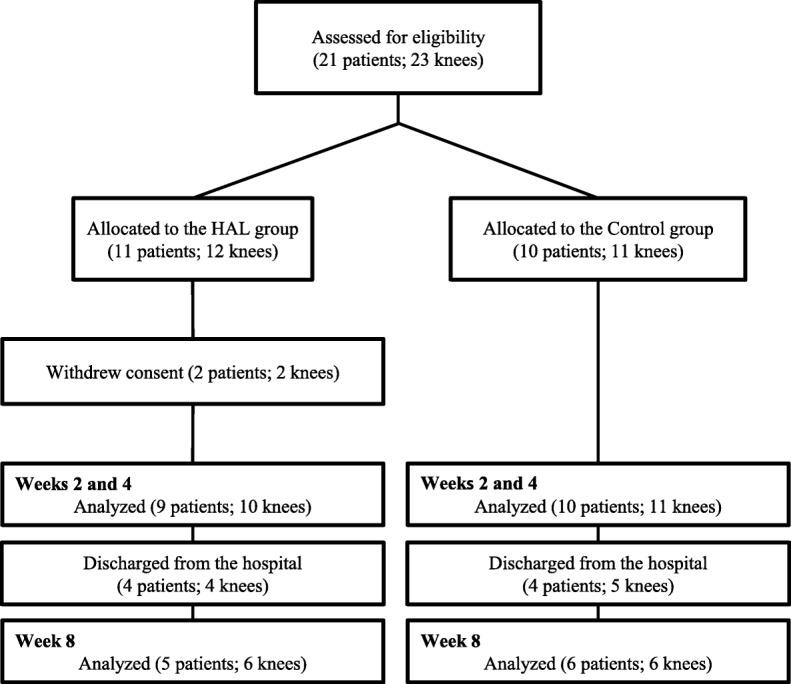
A flowchart of this study

The 9 patients completed the HAL training sessions without adverse events. All 9 patients (10 knees) in the HAL group and 10 patients (11 knees) in the control group were evaluated until week 4. At weeks 8, 4, and 5 patients were discharged from the hospital in the HAL and control groups, respectively (Fig. 2).

The results of walking ability are summarized in Table 3. At weeks 4 and 8, the SWS in the HAL group were greater than in the control group (*P* = 0.030 and *P* = 0.022, respectively). The SWS in the HAL group exceeded the pre-operative value as early as week 4, and only the HAL group showed significantly greater SWS at week 8 than in the pre-operative period (*P* = 0.045) (two-way ANOVA; intervention effect *P =* 0.121 and *η*_*p*_^*2*^ = 0.223; time effect *P <* 0.001 and *η*_*p*_^*2*^ = 0.544; and interactions *P =* 0.304 and *η*_*p*_^*2*^ = 0.112).

**Table 3:** Walking ability in the HAL and control groups

Response		HAL group		Control group			
	Visit	Mean ± SD		Mean ± SD		*P* value	
SWS (m/s)	Pre	1.08 ± 0.29	(*n* = 10)	1.04 ± 0.26	(*n* = 11)	0.794	
	Week 2	0.87 ± 0.19	(*n* = 10)	0.77 ± 0.27	(*n* = 11)	0.366	
	Week 4	1.20 ± 0.09	(*n* = 10)	0.99 ± 0.26	(*n* = 11)	0.030	*
	Week 8	1.34 ± 0.11	(*n* = 6)	1.05 ± 0.23	(*n* = 6)	0.022	*
MWS (m/s)	Pre	1.41 ± 0.33	(*n* = 10)	1.35 ± 0.21	(*n* = 11)	0.588	
	Week 2	1.25 ± 0.38	(*n* = 10)	1.01 ± 0.34	(*n* = 11)	0.137	
	Week 4	1.61 ± 0.32	(*n* = 10)	1.24 ± 0.23	(*n* = 11)	0.006	**
	Week 8	1.63 ± 0.09	(*n* = 6)	1.35 ± 0.24	(*n* = 6)	0.027	*
SL at SWS (m)	Pre	0.59 ± 0.11	(*n* = 10)	0.57 ± 0.11	(*n* = 11)	0.656	
	Week 2	0.54 ± 0.05	(*n* = 10)	0.50 ± 0.09	(*n* = 11)	0.215	
	Week 4	0.63 ± 0.03	(*n* = 10)	0.53 ± 0.07	(*n* = 11)	0.002	**
	Week 8	0.67 ± 0.03	(*n* = 6)	0.56 ± 0.08	(*n* = 6)	0.011	*
SL at MWS (m)	Pre	0.67 ± 0.10	(*n* = 10)	0.62 ± 0.09	(*n* = 11)	0.274	
	Week 2	0.62 ± 0.06	(*n* = 10)	0.52 ± 0.10	(*n* = 11)	0.016	*
	Week 4	0.70 ± 0.05	(*n* = 10)	0.58 ± 0.07	(*n* = 11)	0.001	**
	Week 8	0.73 ± 0.03	(*n* = 6)	0.61 ± 0.07	(*n* = 6)	0.003	**
Cadence at SWS (m)	Pre	108.1 ± 13.7	(*n* = 10)	109.9 ± 11.1	(*n* = 11)		
	Week 2	96.2 ± 13.7	(*n* = 10)	91.5 ± 22.9	(*n* = 11)		
	Week 4	115.1 ± 6.9	(*n* = 10)	111.3 ± 20.7	(*n* = 11)		
	Week 8	119.9 ± 5.3	(*n* = 6)	113.1 ± 13.4	(*n* = 6)		
Cadence at MWS (m)	Pre	125.4 ± 14.9	(*n* = 10)	130.7 ± 11.2	(*n* = 11)		
	Week 2	120.0 ± 25.3	(*n* = 10)	114.0 ± 28.4	(*n* = 11)		
	Week 4	137.7 ± 21.2	(*n* = 10)	127.8 ± 18.1	(*n* = 11)		
	Week 8	133.9 ± 10.0	(*n* = 6)	133.4 ± 20.6	(*n* = 6)		

*Abbreviations*: *SWS*, self-selected walking speed; *MWS*, maximum walking speed; *SL*, step length

*P < 0.05; **P < 0.01

At weeks 4 and 8, the MWS in the HAL group was greater than that in the control group (*P* = 0.006 and *P* = 0.027, respectively) (two-way ANOVA; intervention effect *P =* 0.021 and η_p_^2^ = 0.430; time effect *P =* 0.001 and *η*_*p*_^*2*^ = 0.532; and interactions *P =* 0.111 and *η*_*p*_^*2*^ = 0.198).

The SL at SWS in the HAL group was greater than that in the control group at weeks 4 (*P* = 0.002) and 8 (*P* = 0.011). In the within-group comparison, only the HAL group was found to have significantly greater SL at SWS at weeks 4 and 8 than those at week 2 (*P* = 0.026 and *P* = 0.003, respectively) (two-way ANOVA; intervention effect *P =* 0.048 and *η*_*p*_^*2*^ = 0.337; time effect *P =* 0.017 and *η*_*p*_^*2*^ = 0.341; and interactions *P =* 0.271, *η*_*p*_^*2*^ = 0.123).

The SL at MWS in the HAL group was also greater than that in the control groups at weeks 2 (*P* = 0.016), 4 (*P* = 0.001), and 8 (*P* = 0.003). In the within-group comparison, only the HAL group was found to have significantly greater SL at MWS at week 4 than at week 2 (*P* = 0.010) (two-way ANOVA; intervention effect *P =* 0.003 and *η*_*p*_^*2*^ = 0.594; time effect *P <* 0.001 and *η*_*p*_^*2*^ = 0.586; and interactions *P =* 0.063 and *η*_*p*_^*2*^ = 0.212).

The results of changes in the ROM are summarized in Table 4. At weeks 2 and 4, the passive knee extension ROM was significantly greater in the HAL group than in the control group (*P* = 0.034 and *P* = 0.006, respectively) (two-way ANOVA; intervention effect *P* = 0.135 and *η*_*p*_^*2*^ = 0.209; time effect *P =* 0.071 and *η*_*p*_^*2*^ = 0.238; and interactions *P =* 0.419 and *η*_*p*_^*2*^ = 0.082). At weeks 2 and 3, the active knee extension ROM was significantly greater in the HAL group than in the control group (*P* = 0.005 and *P* = 0.048, respectively) (two-way ANOVA; intervention effect *P* = 0.120 and *η*_*p*_^*2*^ = 0.225; time effect *P* = 0.082 and *η*_*p*_^*2*^ = 0.204; and interactions *P =* 0.262 and *η*_*p*_^*2*^ = 0.124).

**Table 4:** Range of motion in the HAL and control groups

Response		HAL group		Control group			
	Visit	Mean ± SD		Mean ± SD		*P* value	
Knee ROM (degree)							
Passive extension	Pre	− 4.0 ± 8.4	(*n* = 10)	− 6.4 ± 5.0	(*n* = 11)	0.440	
	Week 1	− 5.0 ± 3.3	(*n* = 10)	− 8.6 ± 6.4	(*n* = 11)	0.117	
	Week 2	− 3.0 ± 4.2	(*n* = 10)	− 7.7 ± 5.2	(*n* = 11)	0.034	*
	Week 3	− 2.0 ± 3.5	(*n* = 10)	− 5.9 ± 4.9	(*n* = 11)	0.051	
	Week 4	− 0.5 ± 1.6	(*n* = 10)	-5.5 ± 4.7	(*n* = 11)	0.006	**
	Week 8	− 0.8 ± 2.0	(*n* = 6)	-4.2 ± 3.8	(*n* = 6)	0.086	
Active extension	Pre	− 7.0 ± 5.4	(*n* = 10)	− 6.8 ± 5.6	(*n* = 11)	0.940	
	Week 1	− 10.5 ± 7.6	(*n* = 10)	− 13.2 ± 7.2	(*n* = 11)	0.416	
	Week 2	−5.0 ± 5.3	(*n* = 10)	− 12.3 ± 5.2	(*n* = 11)	0.005	**
	Week 3	− 4.0 ± 3.9	(*n* = 10)	− 7.7 ± 4.1	(*n* = 11)	0.048	*
	Week 4	− 3.5 ± 4.1	(*n* = 10)	-6.4 ± 6.0	(*n* = 11)	0.220	
	Week 8	− 2.5 ± 2.7	(*n* = 6)	-5.8 ± 3.8	(*n* = 6)	0.110	
Passive flexion	Pre	126.0 ± 20.2	(*n* = 10)	119.1 ± 18.4	(*n* = 11)		
	Week 1	95.3 ± 16.9	(*n* = 10)	95.5 ± 7.6	(*n* = 11)		
	Week 2	103.5 ± 11.1	(*n* = 10)	102.7 ± 8.8	(*n* = 11)		
	Week 3	109.5 ± 9.8	(*n* = 10)	108.2 ± 9.3	(*n* = 11)		
	Week 4	115.8 ± 9.2	(*n* = 10)	110.9 ± 10.9	(*n* = 11)		
	Week 8	122.5 ± 11.7	(*n* = 6)	117.8 ± 11.4	(*n* = 6)		
Active flexion	Pre	123.0 ± 22.4	(*n* = 10)	115.0 ± 17.0	(*n* = 11)		
	Week 1	85.5 ± 25.1	(*n* = 10)	85.0 ± 12.2	(*n* = 11)		
	Week 2	96.0 ± 14.3	(*n* = 10)	94.1 ± 10.9	(*n* = 11)		
	Week 3	100.0 ± 21.6	(*n* = 10)	99.5 ± 13.5	(*n* = 11)		
	Week 4	108.0 ± 15.1	(*n* = 10)	104.1 ± 13.0	(*n* = 11)		
	Week 8	115.8 ± 13.6	(*n* = 6)	109.2 ± 11.6	(*n* = 6)		

*P < 0.05; **P < 0.01

The results of muscle strength are summarized in Table 5. The knee extension torque was significantly higher in the HAL group than in the control group at week 8 (*P* = 0.014) (two-way ANOVA; intervention effect *P =* 0.233 and *η*_*p*_^*2*^ = 0.173; time effect *P =* 0.009 and *η*_*p*_^*2*^ = 0.530; and interactions *P =* 0.422 and *η*_*p*_^*2*^ = 0.092).

**Table 5:** Muscle strength in the HAL and control groups

Response		HAL group		Control group			
	Visit	Mean ± SD		Mean ± SD		*P* value	
Knee extension torque (Nm/kg)	Pre	1.10 ± 0.64	(*n* = 10)	0.91 ± 0.31	(*n* = 11)	0.373	
	Week 2	0.68 ± 0.43	(*n* = 10)	0.67 ± 0.23	(*n* = 10)	0.924	
	Week 4	0.93 ± 0.32	(*n* = 10)	0.85 ± 0.23	(*n* = 11)	0.541	
	Week 8	1.15 ± 0.12	(*n* = 6)	0.88 ± 0.17	(*n* = 5)	0.014	*
Knee flex torque (Nm/kg)	Pre	0.56 ± 0.22	(*n* = 10)	0.51 ± 0.22	(*n* = 11)		
	Week 2	0.36 ± 0.15	(*n* = 10)	0.41 ± 0.15	(*n* = 10)		
	Week 4	0.45 ± 0.16	(*n* = 10)	0.41 ± 0.15	(*n* = 11)		
	Week 8	0.51 ± 0.12	(*n* = 6)	0.50 ± 0.15	(*n* = 5)		

*P < 0.05

The results of WOMAC are summarized in Table 6. The WOMAC-P in the HAL group at week 2 was greater than that in the control group (*P* = 0.021), and the lowering of pain was recognized early in the HAL group (two-way ANOVA; intervention effect *P =* 0.336 and *η*_*p*_^*2*^ = 0.103; time effect *P =* 0.002 and *η*_*p*_^*2*^ = 0.427; and interactions *P =* 0.023 and *η*_*p*_^*2*^ = 0.293 in the WOMAC-P, and intervention effect *P =* 0.073 and *η*_*p*_^*2*^ = 0.313; time effect *P =* 0.019 and *η*_*p*_^*2*^ = 0.304; and interactions *P =* 0.109 and *η*_*p*_^*2*^ = 0.198 in the WOMAC-F, respectively).

**Table 6:** Knee pain and functionality in the HAL and control groups

Response		HAL group		Control group			
	Visit	Mean ± SD		Mean ± SD		*P* value	
WOMAC-P	Pre	72.0 ± 13.4	(*n* = 10)	60.0 ± 23.2	(*n* = 11)	0.169	
	Week 2	78.0 ± 15.7	(*n* = 10)	59.1 ± 18.4	(*n* = 11)	0.021	*
	Week 4	79.0 ± 12.4	(*n* = 10)	80.0 ± 13.6	(*n* = 11)	0.863	
	Week 8	88.0 ± 5.7	(*n* = 5)	79.2 ± 14.6	(*n* = 6)	0.218	
WOMAC-F	Pre	82.2 ± 16.9	(*n* = 10)	74.6 ± 15.0	(*n* = 11)	0.287	
	Week 2	82.8 ± 14.5	(*n* = 9)	69.6 ± 19.0	(*n* = 10)	0.110	
	Week 4	86.5 ± 10.6	(*n* = 10)	83.3 ± 11.3	(*n* = 11)	0.515	
	Week 8	92.6 ± 6.6	(*n* = 5)	85.3 ± 5.7	(*n* = 6)	0.077	

*Abbreviations*: Western Ontario and McMaster Universities Osteoarthritis Index *WOMAC-P*, Subscale of pain in WOMAC; *WOMAC-F*, Subscale of function in WOMAC

*P < 0.05

The results of two-way ANOVA in the cadence at SWS and MWS, the passive and active knee flexion ROM and the torque of knee flexion showed a significant effect only in the time effect.

## Discussion

The walking speed in the HAL group was better than that in the control group at weeks 4 and 8. In addition, the SWS of the HAL group exceeded the pre-operative SWS by as early as 4 weeks, and only the HAL group showed significantly better SWS at week 8 than in the pre-operative period. This difference exceeded the minimum clinically meaningful change in walking speed suggested for people with knee pain [31, 32]; thus, clinically meaningful changes were obtained early in the HAL group. Early recovery beyond the pre-operative period allows early discharge and early social reversion. In past studies, the post-operative walking speed did not recover to the pre-operative speed at 4 or 8 weeks postoperatively [33, 34], but was found to recover by 12 weeks [4, 5, 7, 31]. Owing to the early recovery in walking speed, it can be stated that HAL training can be an effective rehabilitation intervention for patients after TKA.

At weeks 2 and 3, the active knee extension ROM in the HAL group was better than that in the control group. Extension lag has been reported to be associated with muscle strain reduction in the quadriceps muscles [9]. Improvement in extension lag at week 2 implies recovery of knee extension, possibly through a potential neuromuscular mechanism [8, 35], and might have led to an improvement in the knee extension torque in the HAL group than in the control group, which was observed at weeks 8. In a case report where a HAL-SJ was applied after TKA [26], the authors suggested that improvement in the extension lag was brought about by the HAL-SJ-mediated promotion of muscle and nerve function in the quadriceps muscle. Due to the assistance during knee motion, the extension lag during walking decreased, and it was believed that the increase in step length led to an improvement in walking speed. Similarly, in our previous study on HAL training in stroke patients, improvements in walking speed accompanied by improvements in gait symmetry and increase in step length were observed [36]. The torque assistance provided by the HAL allows the therapist to gradually adjust the magnitude of the myoelectric potential emitted by the patient in real time, and it allows the adjustment of the degree of effort required by the patient to walk almost normally. The involvement of repetitive movements and voluntary activities is an important factor for motor learning [37, 38]. HAL training for post-TKA patients seems to be a voluntary iterative exercise of sensory feedback of normal joint movement and walking pattern.

The WOMAC-P score was significantly greater in the HAL group than in the control group, indicating that postoperative pain was relieved by early as week 2. Knee pain is associated with abnormal muscle activity [39], co-contraction [40], and weakness [41], and it is known to affect extension lag [10] and walking ability [42]. By using HAL, gradual assistance is provided to patients so that pain is not experienced or aggravated, and exercises are conducted so as not to cause co-contraction of the flexion and extension muscles at an early stage. These may have contributed to the improvement in walking ability and the reduction in extension lag.

In the HAL group, the patients could participate in all sessions without experiencing adverse events. HAL training after TKA can therefore be considered safe. In fact, the time of HAL training is unexpectedly less than the set time limit, and it seems that the cost effectiveness and wearing time of HAL training should be evaluated in future trials.

## Limitations

Although there is no difference between the two groups in terms of patients’ background, this study was non-randomized and non-blinded with a small number of patients. Since the size of single leg HAL was medium only at that time, only the patients who agreed to receive HAL training and their physique allowed for HAL device underwent HAL training. Moreover, there is no data over 8 weeks. Therefore, in the future, a randomized controlled trial with a larger number of patients and longer follow-up is necessary to consider as potential bias risks. However, the intervention procedure and evaluation items in this study are reasonable, and this pilot study can be helpful in planning the future randomized controlled trial. Although there is no significant difference between the two groups in the number of cases that already undergone TKA on the contralateral side and the total physical therapy time including HAL training, it is necessary to align these precisely in the future study.

## Conclusion

HAL training after TKA can improve walking speed, step length, early active knee extension ROM, and muscle strength without severe pain better than conventional rehabilitation for up to 8 weeks after TKA. Since the recovery of walking ability was earlier in the HAL group than in the control group and no adverse events were noted, HAL training can be considered a safe and effective rehabilitation intervention for patients who have undergone TKA. Since this study was a preliminary study, a randomized controlled trial with a larger number of patients and loner follow-up is necessary in the future.

## Abbreviations

ADL: Activities of daily living
ANOVA: Analysis of variance
CVC: Cybernic Voluntary Control
HAL: Hybrid Assistive Limb
MWS: Maximum walking speed
OA: Osteoarthritis
QOL: Quality of life
RA: Rheumatoid arthritis
RAT: Robot-assisted training
ROM: Range of motion
SL-MWS: Step length at maximum walking speed
SL-SWS: Step length at self-selected walking speed
SWS: Self-selected walking speed
TKA: Total knee arthroplasty
WOMAC: Western Ontario and McMaster Universities Osteoarthritis Index
WOMAC-f: Subscale of function in WOMAC
WOMAC-p: Subscale of pain in WOMAC
